# Impact of preoperative psychological status on clinical outcomes following percutaneous endoscopic lumbar surgery under local anesthesia: a propensity score-matched analysis

**DOI:** 10.3389/fpsyg.2026.1904164

**Published:** 2026-08-20

**Authors:** Hanbiao Zhou, Xuan Sun, Bo Huang, Kai Sun

**Affiliations:** 1 Department of Orthopedic Surgery, The Sixth Affiliated Hospital of Jinan University (Dongguan), Dongguan, China; 2 Dongguan Key Laboratory of Central Nervous System Injury and Repair, Dongguan, China; 3 First Clinical Medical College of Yangtze University, Jingzhou, China; 4 Department of Orthopedic Surgery, Jiujiang University Affiliated Hospital, Jiujiang, China

**Keywords:** endoscopic lumbar surgery, local anesthesia, pain catastrophizing, propensity score matching, psychological distress

## Abstract

**Background:**

The application of percutaneous endoscopic lumbar surgery under local anesthesia is becoming increasingly widespread; however, the impact of preoperative psychological status on surgical outcomes remains unclear. This study utilized propensity score matching to evaluate the effects of psychological distress and pain catastrophizing on clinical outcomes.

**Methods:**

A retrospective cohort of 148 propensity-score-matched patients (74 per group) undergoing percutaneous endoscopic lumbar discectomy under local anesthesia was analyzed. Preoperative psychological status was assessed using the Hospital Anxiety and Depression Scale (HADS) and the Pain Catastrophizing Scale (PCS). Outcome measures included the Visual Analog Scale (VAS) for leg and back pain, the Oswestry Disability Index (ODI), the Modified MacNab criteria, and the achievement rate of the Minimal Clinically Important Difference (MCID) at 12 months postoperatively.

**Results:**

At baseline, no significant differences were observed between the distress and non-distress groups in leg VAS (7.6 ± 2.3 vs. 7.5 ± 2.2, *p* = 0.681), back VAS (6.6 ± 1.3 vs. 6.5 ± 1.2, *p* = 0.093), or ODI (54.8 ± 9.5 vs. 53.9 ± 9.7, *p* = 0.594). At 12 months postoperatively, the distress group exhibited significantly higher leg VAS (3.7 ± 1.9 vs. 2.0 ± 1.1, *p* = 0.033), back VAS (3.1 ± 1.5 vs. 2.0 ± 1.0, *p* = 0.024), and ODI (31.2 ± 10.8 vs. 21.0 ± 7.5, *p* = 0.012) scores. The rate of MCID achievement for ODI was significantly lower in the distress group (60.8% vs. 82.4%, *p* = 0.015). Furthermore, the proportion of patients achieving excellent or good outcomes according to the Modified MacNab criteria was 81.1% in the distress group compared to 93.2% in the non-distress group (*p* = 0.021). Both HADS and PCS scores were identified as independent predictors of unsatisfactory outcomes (adjusted OR = 1.17 and 1.09, respectively; both *p* < 0.01). The combined clinical-psychological model demonstrated superior predictive accuracy (AUC = 0.84).

**Conclusion:**

Preoperative psychological distress and pain catastrophizing are independent predictors of inferior clinical outcomes following percutaneous endoscopic lumbar surgery under local anesthesia. Routine preoperative psychological screening and targeted interventions may be beneficial for optimizing surgical outcomes.

## 1 Introduction

Lumbar disc herniation is a major cause of radiculopathy and functional disability worldwide, contributing substantially to global disease burden and healthcare utilization (Vos et al., 2020). With the increasing demand for minimally invasive spine surgery, percutaneous endoscopic lumbar procedures have gained widespread acceptance due to reduced soft tissue damage, shorter hospital stay, and faster postoperative recovery compared with conventional open techniques (Phan et al., 2021; Kotheeranurak et al., 2023; Ahn, 2009).

Unlike traditional spine surgeries performed under general anesthesia, percutaneous endoscopic lumbar surgery is frequently conducted under local anesthesia, which enables real-time intraoperative neurological monitoring through patient feedback (Ruetten et al., 2008). This awake surgical setting has been shown to enhance procedural safety by reducing the risk of nerve injury; however, it also introduces unique challenges, as patients remain conscious throughout the procedure and may experience heightened anxiety, pain sensitivity, and psychological stress (Wang and Grossman, 2002). While direct comparisons between local and general anesthesia have yielded mixed results regarding psychological outcomes (Javeed et al., 2024), the conscious nature of this procedure presents a distinct clinical context in which preoperative psychological factors may play a particularly important role in shaping intraoperative experience and postoperative recovery (Aghajanian et al., 2024; Wagner et al., 2020; Hägg et al., 2003). In parallel, pain catastrophizing—a maladaptive cognitive-emotional response characterized by magnification of pain-related stimuli and feelings of helplessness—has been shown to significantly amplify pain perception and predict poorer postoperative recovery (Yang et al., 2025; Kim et al., 2015). These psychological factors are thought to influence central pain processing through mechanisms such as central sensitization and altered descending inhibitory pathways (Ye et al., 2024).

Despite growing recognition of the role of psychological factors in spine surgery, current evidence is largely derived from open or fusion procedures under general anesthesia (Aghajanian et al., 2024; Petrucci et al., 2025). Studies specifically evaluating minimally invasive endoscopic surgery, particularly under local anesthesia, remain scarce. Therefore, we hypothesized that preoperative psychological distress and pain catastrophizing adversely affect clinical outcomes following percutaneous endoscopic lumbar surgery under local anesthesia. The aim of this study was to evaluate their impact using propensity score-matched analysis and to develop a predictive model for postoperative outcomes.

## 2 Materials and methods

### 2.1 Study design and patient selection

This retrospective cohort study was conducted at a single spine center between January 2020 and December 2024. The study protocol was approved by the institutional review board, and all procedures were performed in accordance with the Declaration of Helsinki. The institutional review board approved the study and waived the requirement for informed consent due to the retrospective nature of the analysis and the use of de-identified data.

Consecutive patients who underwent percutaneous endoscopic lumbar discectomy under local anesthesia for lumbar disc herniation were screened.

#### 2.1.1 Inclusion criteria:

- age 18–65 years;
- single-level lumbar disc herniation confirmed by MRI;
- persistent radicular symptoms refractory to ≥6 weeks of conservative treatment;
- treatment with percutaneous endoscopic lumbar surgery under local anesthesia;
- minimum follow-up of 12 months.

#### 2.1.2 Exclusion criteria:

- prior lumbar spine surgery;
- lumbar instability or spondylolisthesis > Grade I;
- spinal infection, tumor, or fracture;
- severe psychiatric disorders requiring pharmacological treatment;
- incomplete clinical or follow-up data.

### 2.2 Propensity score matching

To reduce selection bias, propensity score matching (PSM) was performed. Propensity scores were estimated using a multivariable logistic regression model with psychological distress status (high vs. low) as the dependent variable. Covariates included age, sex, body mass index (BMI), symptom duration, baseline Visual Analog Scale (VAS) scores, and baseline Oswestry Disability Index (ODI). Patients were matched in a 1:1 ratio using nearest-neighbor matching without replacement, with a caliper width of 0.02 of the standard deviation of the logit of the propensity score. Covariate balance was assessed using standardized mean differences (SMD), with values < 0.1 indicating adequate balance. For regression analysis, the total HADS score (sum of anxiety and depression subscales, range 0–42) was used as a continuous variable to preserve statistical power.

### 2.3 Psychological assessment

Preoperative psychological status was assessed within 1 week prior to surgery using the Hospital Anxiety and Depression Scale (HADS) and the Pain Catastrophizing Scale (PCS). The HADS is a 14-item self-administered instrument comprising two subscales evaluating anxiety (HADS-A) and depression (HADS-D), each scored from 0 to 21. A score ≥8 on either subscale defined clinically significant psychological distress. The PCS total score ranges from 0 to 52, with higher scores indicating greater catastrophic thinking.

### 2.4 Surgical procedure

All procedures were performed under local anesthesia by experienced spine surgeons using a standardized percutaneous endoscopic lumbar technique. Local anesthetic was infiltrated along the planned trajectory. A working cannula was introduced under fluoroscopic guidance via a transforaminal or interlaminar approach. Endoscopic decompression was then performed, including removal of herniated disc material and decompression of the affected neural elements.

### 2.5 Outcome measures and follow-up

**Figure 1:**
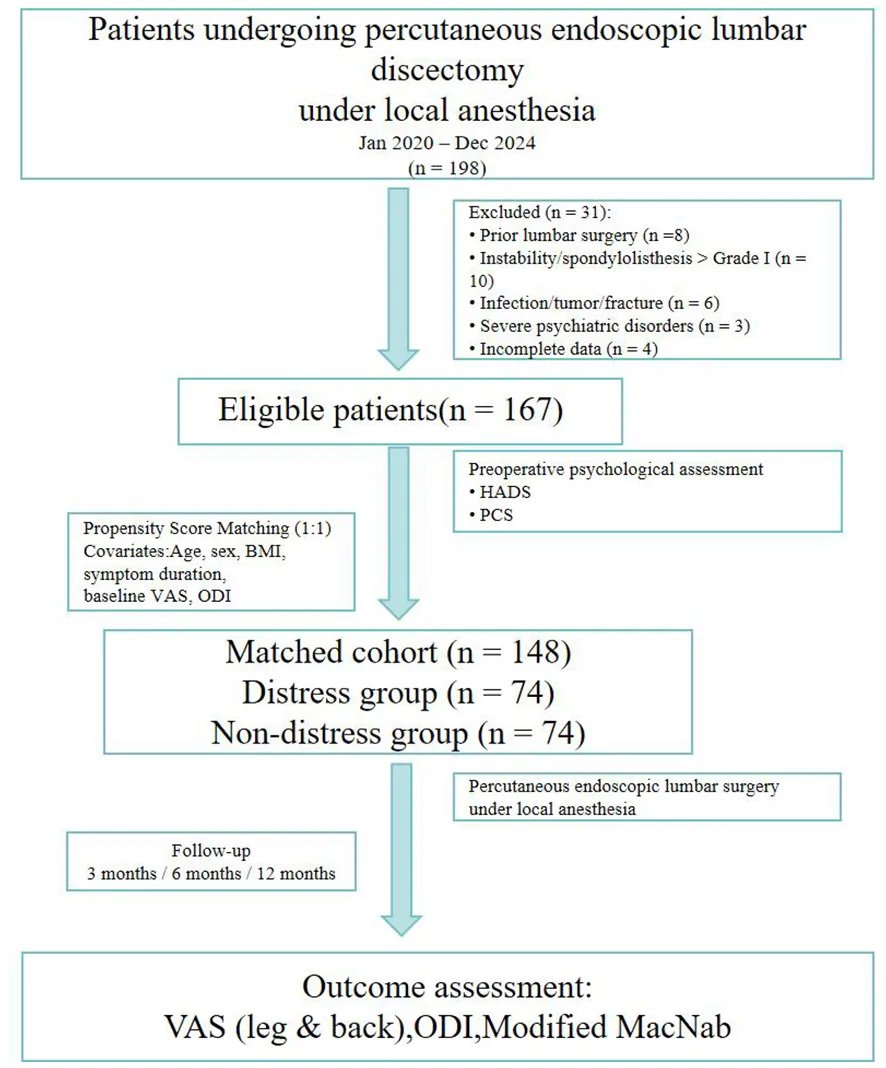
Study flow diagram of patient selection, propensity score matching, and outcome assessment. A total of 198 patients who underwent percutaneous endoscopic lumbar discectomy under local anesthesia between January 2020 and December 2024 were screened. After applying exclusion criteria, 167 eligible patients were included. Patients were stratified into psychological distress and non-distress groups based on preoperative HADS scores. Propensity score matching (1:1) was performed, resulting in a matched cohort of 148 patients (74 per group). All patients underwent standardized surgical procedures and were followed up at 3, 6, and 12 months. Clinical outcomes, including VAS, ODI, Modified MacNab criteria, and MCID achievement, were assessed.

Clinical outcomes were collected at baseline and at 3, 6, and 12 months postoperatively. The primary endpoint was achievement of minimal clinically important difference (MCID).The MCID thresholds were defined as ≥12 points for ODI and ≥2 points for VAS, based on previously published literature. Secondary endpoints included VAS for leg and back pain, ODI scores, Modified MacNab criteria (Figure 1).

### 2.6 Statistical analysis

All statistical analyses were performed using SPSS (version 26.0, IBM Corp.) and R software (version 4.2.1, R Foundation). Propensity score matching was performed as described above. Continuous variables are presented as mean ± SD and were compared using independent-samples t-tests or Mann–Whitney *U* tests; categorical variables are presented as frequencies (%) and were compared using chi-square or Fisher's exact test.To identify independent predictors of unsatisfactory outcome (fair/poor Modified MacNab at 12 months), univariate logistic regression was performed, and variables with *p* < 0.10 were entered into a multivariate model. Results are reported as odds ratios (ORs) with 95% confidence intervals (CIs).

The discriminative ability of the predictive model was evaluated using receiver operating characteristic (ROC) curves and the area under the curve (AUC). A two-tailed *p* < 0.05 was considered statistically significant, with no adjustment for multiple comparisons. Differences between ROC curves were compared using the DeLong test.

## 3 Results

### 3.1 Baseline characteristics after propensity score matching

A total of 198 consecutive patients were initially screened. After applying the exclusion criteria, 167 eligible patients were included in the study. Patients were classified into psychological distress and non-distress groups based on preoperative HADS scores. Propensity score matching (1:1) yielded 148 matched patients (74 per group).As shown in Table 1, there were no significant differences between the two groups after matching in age (47.9 ± 12.4 vs. 47.2 ± 10.8 years, *p* = 0.712), sex (55.4% vs. 54.1% male, p = 0.873), BMI (24.9 ± 4.1 vs. 24.7 ± 5.3 kg/m^2^, p = 0.641), symptom duration >6 months (44.6% vs. 41.9%, *p* = 0.728), or baseline VAS and ODI scores (all *p* > 0.05). All standardized mean differences were < 0.1, indicating adequate covariate balance.

**Table 1:** Baseline characteristics before and after propensity score matching.

Variable	Distress group (matched, *n* = 74)	Non-distress group (matched, *n* = 74)	*p*-value
Age (years)	47.9 ± 12.4	47.2 ± 10.8	0.712
Male, *n* (%)	41 (55.4%)	40 (54.1%)	0.873
BMI (kg/m^2^)	24.9 ± 4.1	24.7 ± 5.3	0.641
Symptom duration >6 months, n (%)	33 (44.6%)	31 (41.9%)	0.728
Baseline VAS-leg	7.6 ± 2.3	7.5 ±2.2	0.681
Baseline VAS-back	6.6 ± 1.3	6.5 ± 1.2	0.093
Baseline ODI	54.8 ± 9.5	53.9 ± 9.7	0.594
L4/5 level, n (%)	39 (52.7%)	38 (51.4%)	0.882

PSM, propensity score matching; BMI, body mass index; VAS, visual analog scale; ODI, Oswestry disability index. ^*^ *p* < 0.05 indicates statistical significance.

### 3.2 Longitudinal clinical outcomes

Both groups improved significantly after surgery, but the distress group consistently showed worse outcomes (Table 2). For leg pain, VAS scores were comparable preoperatively (7.6 ± 2.3 vs. 7.5 ± 2.2, *p* = 0.681). At 3, 6, and 12 months, the distress group had significantly higher VAS-leg (*p* = 0.008, 0.004, and 0.033, respectively). The 12-month improvement in VAS-leg was lower in the distress group (3.9 ± 2.1 vs. 5.4 ± 1.8, *p* = 0.003).

**Table 2:** Clinical outcomes after propensity score matching.

Outcome	Distress group (*n* = 74)	Non-distress group (*n* = 74)	*p*-value
VAS-leg			
Preoperative	7.5 ±2.2	7.6 ± 2.3	0.681
3 months	3.6 ± 1.7	2.2 ± 1.4	0.008^*^
6 months	3.0 ± 1.6	2.1 ± 1.2	0.004^*^
12 months	3.7 ± 1.9	2.0 ± 1.1	0.033^*^
Improvement (12m)	3.9 ± 2.1	5.4 ± 1.8	0.003^*^
VAS-back			
Preoperative	6.5 ± 1.2	6.6 ± 1.3	0.093
3 months	3.2 ± 1.5	3.8 ± 1.6	0.308
6 months	2.9± 1.8	1.8 ± 1.4	0.015^*^
12 months	3.1 ± 1.5	2.0 ± 1.0	0.024^*^
Improvement (12m)	2.6 ± 1.8	4.4 ± 1.8	0.001^*^
ODI (%)			
Preoperative	53.9 ± 9.7	54.8 ± 9.5	0.594
3 months	35.2 ± 10.3	28.6 ± 8.9	0.026^*^
6 months	30.1 ± 9.7	22.4 ± 7.8	0.034^*^
12 months	31.2 ± 10.8	21.0 ± 7.5	0.012^*^
Improvement rate (%)	43.39%	61.25%	0.005^*^
**Modified MacNab (12 months)**			
Excellent/good	60 (81.1%)	69 (93.2%)	0.021^*^
Fair/poor	14 (18.9%)	5 (6.8%)	

VAS, visual analog scale; ODI, oswestry disability index. Improvement rate (%) = (preoperative-−12-month ODI) / preoperative ODI × 100. ^*^ *p* < 0.05 indicates statistical significance.

For back pain, no significant difference was observed at 3 months (*p* = 0.308), but at 6 and 12 months the distress group had higher VAS-back (*p* = 0.015 and 0.024). The 12-month improvement in VAS-back was also lower (2.6 ± 1.8 vs. 4.4 ± 1.8, *p* = 0.001).Regarding functional disability (ODI), baseline scores were similar (*p* = 0.594). At 3, 6, and 12 months, the distress group had significantly higher ODI scores (*p* = 0.026, 0.034, and 0.012). The improvement rate at 12 months was 43.39% in the distress group vs. 61.25% in the non-distress group (*p* = 0.005).

### 3.3 Global outcome and MCID achievement

At 12 months, the Modified MacNab criteria showed that the non-distress group achieved excellent/good outcomes significantly more often (93.2% vs. 81.1%, *p* = 0.021) (Table 2). As presented in Table 3, the distress group had significantly lower MCID achievement rates for VAS-leg (70.3% vs. 87.8%, *p* = 0.034), VAS-back (70.3% vs. 87.8%, *p* = 0.028), and ODI (60.8% vs. 82.4%, *p* = 0.015). The proportion achieving both VAS and ODI MCID was 55.4% in the distress group vs. 78.4% (*p* = 0.006).

**Table 3:** Comparison of MCID Achievement rates at 12 months after PSM.

Outcome	Distress group (*n* = 74)	Non-distress group (*n* = 74)	*p*-value
VAS-leg MCID achieved	52 (70.3%)	65 (87.8%)	0.034^*^
VAS-back MCID achieved	52 (70.3%)	65 (87.8%)	0.028^*^
ODI MCID achieved	45 (60.8%)	61 (82.4%)	0.015^*^
Both VAS and ODI MCID achieved	41 (55.4%)	58 (78.4%)	0.006^*^

MCID, minimal clinically important difference. VAS, visual analog scale; ODI, oswestry disability index. ^*^ *p* < 0.05 indicates statistical significance.

### 3.4 Predictors of unsatisfactory outcome

**Figure 2:**
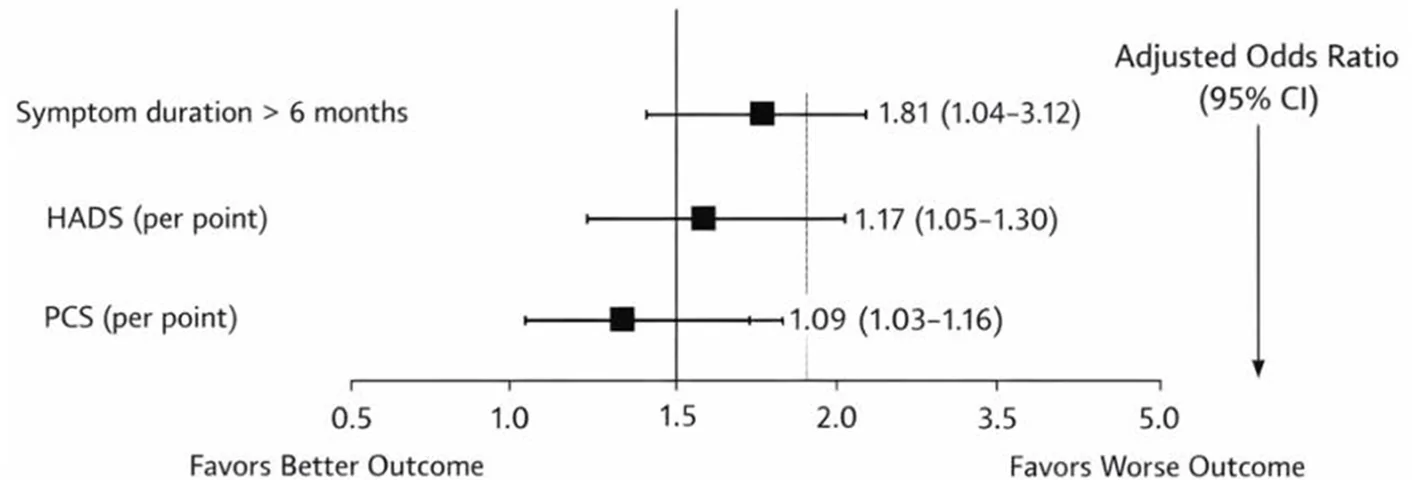
Forest plot of multivariate logistic regression analysis for predictors of unsatisfactory outcome at 12 months. Adjusted odds ratios (ORs) and 95% confidence intervals (CIs) are presented for independent predictors identified in multivariate analysis, including symptom duration >6 months, HADS score, and PCS score. The vertical reference line indicates OR = 1. Variables were selected based on univariate analysis (*p* < 0.10).

Multivariate logistic regression (Table 4) identified symptom duration >6 months (adjusted OR = 1.81, 95% CI: 1.04–3.12, *p* = 0.038), higher HADS score (adjusted OR = 1.17 per point, 95% CI: 1.05–1.30, *p* = 0.004), and higher PCS score (adjusted OR = 1.09 per point, 95% CI: 1.03–1.16, *p* = 0.002) as independent predictors of unsatisfactory outcome (fair/poor Modified MacNab) at 12 months. Figure 2 shows the forest plot of these adjusted odds ratios.

**Table 4:** Univariate and multivariate logistic regression analysis for unsatisfactory outcome at 12 months (After PSM).

Variable	Univariate OR (95% CI)	*p*-value	Multivariate β	SE	Adjusted OR (95% CI)	*p*-value
Age (per year)	1.02 (0.98–1.06)	0.312	—	—	—	—
Male sex	0.89 (0.41–1.94)	0.771	—	—	—	—
BMI (per kg/m^2^)	1.05 (0.94–1.17)	0.394	—	—	—	—
Symptom duration >6 months	2.14 (1.08–4.23)	0.029^*^	0.593	0.287	1.81 (1.04–3.12)	0.038^*^
Baseline VAS-leg	1.16 (0.89–1.51)	0.274	—	—	—	—
Baseline VAS-Back	1.05 (0.85–1.30)	0.274	—	—	—	—
Baseline ODI	1.03 (0.99–1.06)	0.118	—	—	—	—
HADS score (per point)	1.21 (1.09–1.34)	< 0.001^*^	0.157	0.054	1.17 (1.05–1.30)	0.004^*^
PCS score (per point)	1.13 (1.06–1.20)	< 0.001^*^	0.086	0.028	1.09 (1.03–1.16)	0.002^*^

Outcome variable: unsatisfactory outcome (fair/poor Modified MacNab) at 12 months. Variables with *p* < 0.10 in univariate analysis were entered into multivariate logistic regression. OR, odds ratio; CI, confidence interval; SE, standard error; HADS, hospital anxiety and depression scale; PCS, pain catastrophizing scale. ^*^ *p* < 0.05 indicates statistical significance.

### 3.5 Predictive model performance

**Figure 3:**
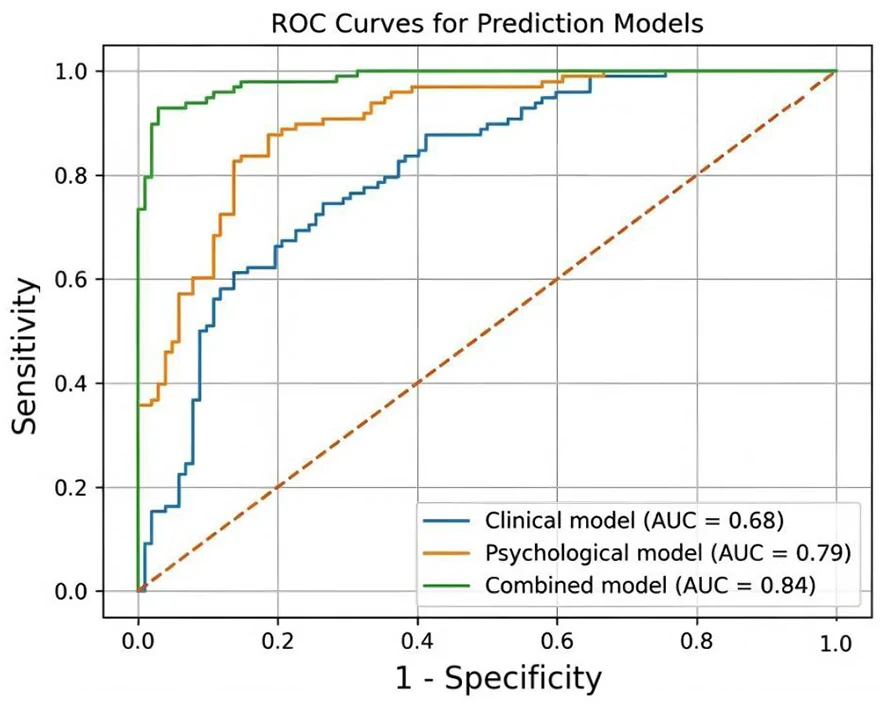
Receiver operating characteristic (ROC) curves for prediction models. The clinical model demonstrated moderate discriminative ability (AUC = 0.68), while the psychological model showed improved performance (AUC = 0.79 ). The combined model achieved the highest predictive accuracy (AUC = 0.84 ). Differences between ROC curves were compared using the DeLong test.

ROC curve analysis (Figure 3) demonstrated that the clinical model (only clinical variables) had moderate discriminative ability (AUC = 0.68). The psychological model (HADS and PCS) showed substantially improved performance (AUC = 0.79). The combined model, integrating both clinical and psychological variables, achieved the highest predictive accuracy (AUC = 0.84), indicating significant incremental prognostic value.

## 4 Discussion

### 4.1 Principal findings

The data presented here reinforce the notion that preoperative psychological status significantly influences the trajectory of recovery following percutaneous endoscopic lumbar surgery. Patients categorized as having high psychological distress not only reported worse pain relief but also exhibited lower functional gains and satisfaction at the 12-month mark. This observation held true across multiple metrics, including VAS scores, ODI, and the Modified MacNab criteria, suggesting a systemic rather than isolated effect (Javeed et al., 2024; Aghajanian et al., 2024; Wagner et al., 2020).

One intriguing observation in our longitudinal data was the transient crossover in back pain scores at the 3-month follow-up. Although the non-distress group had a slightly higher mean VAS-back score (3.8 ± 1.6 vs. 3.2 ± 1.5), this difference was not statistically significant (*p* = 0.308). Nevertheless, this early trend warrants explanation. First, a minor baseline imbalance existed (non-distress: 6.6 ± 1.3 vs. distress: 6.5 ± 1.2, *p* = 0.093), and regression to the mean may have contributed to the observed numerical difference at 3 months. Second, patients without psychological distress typically hold more optimistic recovery expectations and may engage in more intensive early physical activities and rehabilitation exercises. At 3 months postoperatively—a period when local inflammation and tissue healing are still ongoing—such increased activity could temporarily exacerbate paraspinal muscle fatigue and localized discomfort, manifesting as marginally higher back pain scores (Eubanks et al., 2023). In contrast, patients with higher distress and catastrophizing tendencies often exhibit fear-avoidance behaviors, leading them to restrict physical movement in the early phase, which may transiently reduce activity-induced pain (Vlaeyen and Linton, 2021). However, this initial avoidance pattern is maladaptive in the long run, as it promotes muscle deconditioning, functional disuse, and sustained central sensitization, ultimately resulting in worse back pain outcomes at 6 and 12 months (Hodges and Smeets, 2015). Thus, the early crossover does not negate the detrimental impact of psychological distress but rather highlights its dynamic and delayed influence on the recovery trajectory.

### 4.2 Comparison with previous studies

The negative impact of psychological factors on spine surgery outcomes has been increasingly recognized. The association between psychological distress and poor surgical outcomes is well-documented in the literature, particularly for open or fusion surgeries performed under general anesthesia (Petrucci et al., 2025; Rathbone et al., 2023). However, our findings extend this knowledge to the specific context of minimally invasive, endoscopic procedures under local anesthesia.

Recent investigations into minimally invasive techniques have yielded heterogeneous results regarding psychological factors (Patel et al., 2024). Our study highlights the importance of psychological assessment in the specific context of awake endoscopic surgery under local anesthesia. Although direct evidence comparing the psychological impact of local vs. general anesthesia in this setting remains inconclusive (Liu et al., 2024), the conscious nature of the procedure means that patients remain aware of the surgical environment throughout, which may heighten their perception of intraoperative stressors and nociception(Woolf, 2020). Nevertheless, we acknowledge that our study design—which included only patients undergoing local anesthesia—does not permit direct comparisons with general anesthesia, and our findings should be interpreted within this context.

### 4.3 Potential mechanisms

Several mechanisms may explain the observed association. Several interconnected mechanisms likely explain these findings. First, the phenomenon of central sensitization must be considered (Tracey and Mantyh, 2021). Patients with anxiety or depression often display dysregulation in their descending inhibitory pathways. This neurophysiological state can amplify pain perception, meaning that even successful mechanical decompression may not translate to pain relief if the central nervous system remains hyperexcitable (Quartana et al., 2020). Second, pain catastrophizing functions as a cognitive amplifier. Patients who magnify pain stimuli or feel helpless are more likely to engage in pain-related rumination and avoidance behaviors, which are known to hinder functional recovery (Sullivan et al., 2021; Kehlet et al., 2006). Lastly, the physiological stress response cannot be ignored. Psychological distress may dysregulate the hypothalamic-pituitary-adrenal (HPA) axis, fostering an inflammatory environment that impedes optimal healing (Slavich and Irwin, 2014).

### 4.4 Clinical implications

Unlike conventional lumbar decompression or fusion procedures performed under general anesthesia, percutaneous endoscopic lumbar surgery under local anesthesia represents a unique clinical scenario in which patients remain conscious throughout the operation. In this setting, psychological factors may influence not only postoperative recovery but also the entire perioperative experience. Anxiety, fear, and pain catastrophizing may increase intraoperative pain perception, reduce patient cooperation, and negatively affect procedural tolerance, which may subsequently influence postoperative satisfaction and functional recovery (Deyo, 2010). Therefore, identifying patients with significant psychological distress before surgery has practical implications beyond outcome prediction, as it may facilitate individualized perioperative management. Furthermore, the awake nature of endoscopic surgery provides an opportunity for targeted psychological optimization that is less applicable to conventional surgery under general anesthesia. Patients identified as being at high psychological risk can receive preoperative counseling, cognitive-behavioral interventions, enhanced education regarding the surgical procedure, or individualized perioperative communication strategies. These relatively low-cost interventions may improve patient cooperation during surgery, reduce perioperative stress responses, and ultimately enhance clinical outcomes without altering the surgical technique itself (Louw et al., 2011). Another important clinical implication is patient selection. Because percutaneous endoscopic lumbar surgery under local anesthesia requires active patient participation and communication throughout the procedure, psychological readiness should be considered alongside conventional radiographic and neurological assessments (Copay et al., 2008; Kim et al., 2018). Incorporating validated instruments such as the Hospital Anxiety and Depression Scale (HADS) and the Pain Catastrophizing Scale (PCS) into routine preoperative evaluation may improve risk stratification and support shared decision-making when selecting the optimal surgical approach.Taken together, our findings suggest that psychological assessment should be regarded as an integral component of perioperative evaluation for awake endoscopic lumbar surgery, rather than merely an adjunctive measure. This represents the principal clinical contribution of the present study and extends current evidence from anatomical risk assessment toward a more comprehensive biopsychosocial model of patient management.

### 4.5 Strengths and limitation

A key strength of this study is its specific focus on an underexplored clinical niche: endoscopic surgery under local anesthesia. The use of propensity score matching also strengthens the internal validity by minimizing selection bias. However, limitations are present. The retrospective, single-center design may affect generalizability. Future prospective studies with larger, multicenter cohorts and longitudinal psychological assessments are necessary to validate these findings. Then, the lack of postoperative psychological reassessment (e.g., HADS and PCS at 12 months) limits our ability to establish causal direction. While our findings demonstrate a strong association between baseline psychological distress and worse outcomes, we cannot definitively determine whether preoperative psychological factors independently drive poor recovery, or whether successful surgical pain relief would have naturally improved psychological status over time. It is plausible that a bidirectional relationship exists—with psychological distress impairing recovery, and persistent pain reinforcing distress. Future prospective studies with serial psychological assessments are needed to elucidate the temporal and causal relationships between psychological status and surgical outcomes.

## 5 Conclusion

Preoperative psychological distress and pain catastrophizing are significant predictors of poorer clinical outcomes following percutaneous endoscopic lumbar surgery under local anesthesia. These findings highlight the importance of incorporating psychological assessment into perioperative management and suggest that targeted psychological interventions may improve surgical outcomes in minimally invasive spine procedures.

## Data Availability

The raw data supporting the conclusions of this article will be made available by the authors, without undue reservation.

## References

[B1] Aghajanian S. Shafiee A. Teymouri Athar M. M. Mohammadifard F. Goodarzi S. Esmailpur F. . (2024). Impact of depression on postoperative medical and surgical outcomes in spine surgeries: a systematic review and meta-analysis. J. Clin. Med. 13:3247. doi: 10.3390/jcm13113247 38892958 PMC11172961

[B2] Ahn Y. (2009). Percutaneous endoscopic lumbar discectomy: technical evolution and clinical outcomes. Neurospine 19, 247–258. doi: 10.1186/1749-799X-4-20 19555483 PMC2712454

[B3] Copay A. G. Glassman S. D. Subach B. R. Berven S. Schuler T. C. Carreon L. Y. (2008). Minimum clinically important difference in lumbar spine surgery patients: a choice of methods using the oswestry disability index, medical outcomes study questionnaire short form 36, and pain scales. Spine J. 8, 968–974. doi: 10.1016/j.spinee.2007.11.006 18201937

[B4] Deyo R. A. (2010). Trends, major medical complications, and charges associated with surgery for lumbar spinal stenosis in older adults. Jama 306, 1259–1265. doi: 10.1001/jama.2011.1300 20371784 PMC2885954

[B5] Eubanks J. E. Carlesso C. Sundaram M. Bejarano G. Smeets R. J. E. M. Skolasky R. . (2023). Prehabilitation for spine surgery: a scoping review. PMandR 15, 1335–1350. doi: 10.1002/pmrj.12956 36730164

[B6] Hägg O. Fritzell P. Ekselius L. Nordwall A. (2003). Predictors of outcome in fusion surgery for chronic low back pain. a report from the Swedish Lumbar Spine study. Eur. Spine J. 12, 22–33. doi: 10.1007/s00586-002-0465-z 12592544

[B7] Hodges P. W. Smeets R. J. (2015). Interaction between pain, movement, and physical activity: a clinical perspective. J. Orthop. Sports Phys. Ther. 50, 418–424. doi: 10.1097/AJP.0000000000000098 24709625

[B8] Javeed S. Benedict B. Yakdan S. Saleem S. Zhang J. K. Botterbush K. . (2024). Implications of preoperative depression for lumbar spine surgery outcomes: a systematic review and meta-analysis. JAMA network open 7:e2348565. doi: 10.1001/jamanetworkopen.2023.48565 38277149 PMC10818221

[B9] Kehlet H. Jensen T. S. Woolf C. J. (2006). Persistent postsurgical pain: risk factors and prevention. Lancet 387, 1853–1862. doi: 10.1016/S0140-6736(06)68700-X 16698416

[B10] Kim H. J. Cho C. H. Kang K. T. Chang B. S. Lee C. K. Yeom J. S. (2015). The significance of pain catastrophizing in clinical manifestations of patients with lumbar spinal stenosis: mediation analysis with bootstrapping. Spine J. 15, 238–246. doi: 10.1016/j.spinee.2014.09.002 25239153

[B11] Kim H. J. Kwon O. H. Chang B. S. Lee C. K. Chun H. J. Yeom J. S (2018). Change in pain catastrophizing in patients with lumbar spinal surgery change of catastrophizing after surgery 1 change in pain catastrophizing in patients with lumbar spinal surgery. Spine J. 18, 115–121. doi: 10.1016/j.spinee.2017.06.028 28669860

[B12] Kotheeranurak V. Liawrungrueang W. Quillo-Olvera J. Siepe C. J. Li Z. Z. Lokhande P. V. . (2023). Full-endoscopic lumbar discectomy approach selection: a systematic review and proposed algorithm. Spine 48, 534–544. doi: 10.1097/BRS.0000000000004589 36745468

[B13] Liu W. Liu L. Pan Z. Gu E. (2024). Percutaneous endoscopic interlaminar discectomy with patients' participation: better postoperative rehabilitation and satisfaction. J. Orthop. Surg. Res. 19:547. doi: 10.1186/s13018-024-05043-w 39237977 PMC11378537

[B14] Louw A. Diener I. Butler D/ S. Puentedura E. J. (2011). The effect of neuroscience education on pain, disability, anxiety, and stress in chronic musculoskeletal pain. Arch. Phys. Med. Rehabil. 92, 2041–2056. doi: 10.1016/j.apmr.2011.07.198 22133255

[B15] Patel M. R. Jacob K. C. Hartman T. J. Nie J. W. Shah V. P. Chavez F. A. . (2024). Patient satisfaction following lumbar decompression: what is the role of mental health?. World Neurosurg. 164, e540–e547. doi: 10.1016/j.wneu.2022.05.017 35568123

[B16] Petrucci G. Papalia G. F. Ambrosio L. Russo F. Marchetti A. De Marinis M. G. . (2025). The influence of psychological factors on postoperative clinical outcomes in patients undergoing lumbar spine surgery: a systematic review and meta-analysis. Eur. Spine J. 34, 1409–1419. doi: 10.1007/s00586-025-08733-z 39988610

[B17] Phan K. Xu J. Schultz K. Alvi M. A. Lu V. M. Kerezoudis P. . (2021). Full-endoscopic vs. microendoscopic lumbar discectomy: a systematic review and meta-analysis. Spine. 46, E256–E267. doi: 10.1016/j.spinee.2025.12.016 41512930

[B18] Quartana P. J. Campbell C. M. Edwards R. R. (2020). Pain catastrophizing: a critical review. Pain 160, S22–S29. doi: 10.1586/ern.0934. 19402782 PMC2696024

[B19] Rathbone J. Rackham M. Nielsen D. Lee S. M. Hing W. Riar S. . (2023). A systematic review of anterior lumbar interbody fusion (ALIF) vs posterior lumbar interbody fusion (PLIF), transforaminal lumbar interbody fusion (TLIF), posterolateral lumbar fusion (PLF). Eur. Spine J. 32, 1911–1926. doi: 10.1007/s00586-023-07567-x 37071155

[B20] Ruetten S. Komp M. Merk H. Godolias G. (2008). Full-endoscopic interlaminar and transforaminal lumbar discectomy vs. conventional microsurgical technique: a prospective, randomized, controlled study. Eur. Spine J. 30, 2196–2206. doi: 10.1097/BRS.0b013e31816c8af7 18427312

[B21] Slavich G. M. Irwin M. R. (2014). From stress to inflammation and major depressive disorder: a social signal transduction theory of depression. Nat. Rev. Immunol. 20, 287–299. doi: 10.1037/a0035302 24417575 PMC4006295

[B22] Sullivan M. J. Thorn B. Haythornthwaite J. A. Keefe F. Martin M. Bradley L. A. . (2021). Theoretical perspectives on the relation between catastrophizing and pain. J Pain 22, 777–790. doi: 10.1097/00002508-200103000-00008 11289089

[B23] Tracey I. Mantyh P. W. (2021). The cerebral signature for pain perception and its modulation. Nat Rev Neurol. 17, 289–302. doi: 10.1016/j.neuron.2007.07.012 17678852

[B24] Vlaeyen J. W. S. Linton S. J. (2021). Fear-avoidance model of chronic musculoskeletal pain: 12 years on. Pain 153, 1144–1147. doi: 10.1016/j.pain.2011.12.009 22321917

[B25] Vos T. Lim S. S. Abbafati C. Abbas K. M. Abbasi M. Abbasifard M. . (2020). Global burden of 369 diseases and injuries in 204 countries and territories, 1990–2019: a systematic analysis for the global burden of disease study 2019. Lancet 396, 1204–1222. doi: 10.1016/S0140-6736(20)30925-9 33069326 PMC7567026

[B26] Wagner A. Shiban Y. Wagner C. Aftahy K. Joerger A. K. Meyer B. . (2020). Psychological predictors of quality of life and functional outcome in patients undergoing elective surgery for degenerative lumbar spine disease. Eur. Spine J. 29, 349–359. doi: 10.1007/s00586-019-06106-x 31414288

[B27] Wang M. Y. Grossman J. (2002). Awake minimally invasive spine surgery: a systematic review of the literature. Neurosurgery 90, 521–529. doi: 10.1007/s 10143- 014-0565-3

[B28] Woolf C. J. (2020). Central sensitization: implications for the diagnosis and treatment of pain. Pain 161, S2–S10.doi: 10.1016/j.pain.09030 20961685 PMC3268359

[B29] Yang E. Schonfeld E. Boyett D. Mummaneni P. V. Chou D. Bydon M. . (2025). Does back pain catastrophizing influence 60-month surgical outcomes for patients with degenerative lumbar spondylolisthesis? a quality outcomes database study: presented at the AANS/CNS joint section on disorders of the spine and peripheral nerves. J. Neurosurg. Spine 1, 1–11. doi: 10.3171/2025.5.SPINE25310 40779803

[B30] Ye L. Lin S. Lv Y. Ge C. Chen X. (2024). The association between mental disorders and postoperative outcomes of scoliosis surgery: a systematic review and meta-analysis. Alpha Psychiatry 25, 142–149. doi: 10.5152/alphapsychiatry.2024, 231431 38798805 PMC11117427

